# Correction: Drago et al. Bacteria Living in Biofilms in Fluids: Could Chemical Antibiofilm Pretreatment of Culture Represent a Paradigm Shift in Diagnostics? *Microorganisms* 2024, *12*, 259

**DOI:** 10.3390/microorganisms12122577

**Published:** 2024-12-13

**Authors:** Lorenzo Drago, Andrea Fidanza, Alessio Giannetti, Alessio Ciuffoletti, Giandomenico Logroscino, Carlo Luca Romanò

**Affiliations:** 1 Laboratory of Clinical Microbiology, Department of Biomedical Sciences for Health, University of Milan, 20133 Milan, Italy; 2 UOC Laboratory of Clinical Medicine, MultiLab Department, IRCCS Multimedica, 20138 Milan, Italy; 3 Mininvasive Orthopaedic Surgery—Department of Life, Health and Environmental Sciences, University of L’Aquila, 67100 L’Aquila, Italy; andrea.fidanza@univaq.it (A.F.); giandomenico.logroscino@univaq.it (G.L.); 4 Unit of Orthopaedics and Traumatology, “SS Filippo e Nicola” Hospital, 67051 Avezzano, Italy; 5 Unit of Orthopaedics and Traumatology, “G. Mazzini” Hospital, 64100 Teramo, Italy; alessio.giannetti@graduate.univaq.it (A.G.); alessio.ciuffoletti@aslteramo.it (A.C.); 6 Romano Institute, Rruga Deshmoret e 4 Shkurtit, 1001 Tirana, Albania; info@romanoinstitute.com

In the original publication [1], “Bidossi, A.; Bottagisio, M.; Savadori, P.; De Vecchi, E. Identification and Characterization of Planktonic Biofilm-Like Aggregates in Infected Synovial Fluids from Joint Infections. *Front. Microbiol*. **2020**, *11*, 1368. https://doi.org/10.3389/fmicb.2020.01368. PMID: 32714301; PMCID: PMC7344156.” was not cited. The citation has now been inserted in Figure 1’s title and in the reference list.

The corrected title for Figure 1 appears below:

**Figure 1.** Bacteria organized in biofilm in synovial fluid. The co-aggregation of numerous bacteria (green dots) can be seen, as outlined by the red line (adapted from Bidossi et al. [25]).

This reference was also added to the reference list as [25]. The order of some of the references has been adjusted accordingly.

The authors state that the scientific conclusions are unaffected. This correction was approved by the Academic Editor. The original publication has also been updated.
